# Indocyanine Green-Camptothecin Co-Loaded Perfluorocarbon Double-Layer Nanocomposite: A Versatile Nanotheranostics for Photochemotherapy and FDOT Diagnosis of Breast Cancer

**DOI:** 10.3390/pharmaceutics13091499

**Published:** 2021-09-17

**Authors:** Yu-Hsiang Lee, Po-Wei Kuo, Chun-Ju Chen, Chu-Jih Sue, Ya-Fen Hsu, Min-Chun Pan

**Affiliations:** 1 Department of Biomedical Sciences and Engineering, National Central University, Taoyuan City 320317, Taiwan; b35894814@gmail.com (P.-W.K.); chen107827012@g.ncu.edu.tw (C.-J.C.); lio10025149@gmail.com (C.-J.S.); 2 Department of Chemical and Materials Engineering, National Central University, Taoyuan City 320317, Taiwan; 3 Department of Surgery, Landseed International Hospital, Taoyuan City 324609, Taiwan; hsuyf@landseed.com.tw; 4 Department of Mechanical Engineering, National Central University, Taoyuan City 320317, Taiwan

**Keywords:** breast cancer, indocyanine green, camptothecin, perfluorocarbon, double emulsion, photochemotherapy, fluorescence diffuse optical tomography

## Abstract

Breast cancer remains the most frequently diagnosed cancer and is the leading cause of neoplastic disease burden for females worldwide, suggesting that effective therapeutic and/or diagnostic strategies are still urgently needed. In this study, a type of indocyanine green (ICG) and camptothecin (CPT) co-loaded perfluorocarbon double-layer nanocomposite named ICPNC was developed for detection and photochemotherapy of breast cancer. The ICPNCs were designed to be surface modifiable for on-demand cell targeting and can serve as contrast agents for fluorescence diffuse optical tomography (FDOT). Upon near infrared (NIR) irradiation, the ICPNCs can generate a significantly increased production of singlet oxygen compared to free ICG, and offer a comparable cytotoxicity with reduced chemo-drug dosage. Based on the results of animal study, we further demonstrated that the ICPNCs ([ICG]/[CPT] = 40-/7.5-μM) in association with 1-min NIR irradiation (808 nm, 6 W/cm^2^) can provide an exceptional anticancer effect to the MDA-MB-231 tumor-bearing mice whereby the tumor size was significantly reduced by 80% with neither organ damage nor systemic toxicity after a 21-day treatment. Given a number of aforementioned merits, we anticipate that the developed ICPNC is a versatile theranostic nanoagent which is highly promising to be used in the clinic.

## 1. Introduction

Breast cancer remains one of the most serious health dilemmas worldwide and is considered as the leading cause of neoplastic disease burden for females, with approximately 1 in 20 people globally and 1 in 8 people in high-income countries being affected [1]. Although technologies of cancer diagnosis and treatment have been dramatically advanced over recent decades, the morbidity and mortality of breast cancer continuously raise that count, with about 24% new cases and a 15% increase in cancer deaths globally in 2018 [2], foreseeing that the incidence will increase to >46% by 2040 according to the GLOBOCAN Cancer Tomorrow prediction tool (https://gco.iarc.fr/tomorrow/en; accessed on 1 May 2021). These circumstances indicate that an improved breast cancer diagnostic and/or an effective therapeutic strategy are still urgently needed.

Fluorescence diffuse optical tomography (FDOT) combining fluorescence molecular tomography (FMT) and DOT has been recognized as an emerging optical imaging technology for localization and quantification of fluorescent targets in vivo. A successful FDOT critically relies on the quantity and species of fluorescent probes that bind to the objective in deep tissue [3]. Different from DOT using endogenous contrast such as hemoglobin, water, and/or lipids for diagnosis [4,5], FDOT employs exogenous fluorophores to enhance tissue contrast since fluorescence signals can provide greater detection sensitivity and specificity compared to endogenous absorption signals [3] and may also be able to assess the microenvironmental conditions around the tissue such as oxygen pressure, pH, and intracellular calcium concentration [6,7,8]. In terms of the application of FDOT in tumor detection, the increased blood flow due to tumor neovascularization and the accumulated fluorophores in the neoplastic tissue may cause a higher optical contrast between the diseased and healthy tissues that is favorable for construction of tumor images. Based on aforementioned merits together with its noninvasive nature, FDOT has been widely examined for broad neoplastic detection such as prostate cancer, lung cancer, brain cancer, and breast cancer [9,10,11,12,13].

Camptothecin (CPT) is one of the US FDA-approved anticancer drugs and has been widely applied to a broad spectrum of cancers including rectal/colon cancer, liver cancer, lung cancer, and breast cancer [14,15,16,17]. The antitumor mechanism of CPT can block the synthesis of topoisomerase I, an enzyme highly related to cell division and thereby damage DNA and prevent the growth of cancer cells accordingly [18]. However, similar to most chemotherapeutic agents, application of CPT is hindered by its inherent deficiencies such as water insolubility, low biocompatibility, insufficient retention at the tumor site, and off-target systemic toxicity [19], implying that mono-drug treatment remains lacking for cancer therapy. 

Combination therapy through co-administration of anticancer agents and/or approaches has long been considered as a promising strategy to improve therapeutic efficacy as well as to minimize side effects. Incorporation of therapeutic methods/agents to decrease the dose of each drug involved may concomitantly suppress cancer development with reduced chemotoxicity, leading to an improved clinical outcome [20,21]. Among various anticancer adjuvants, near infrared (NIR)-based phototherapy has gained increasing attention because (1) NIR may provide increased tissue penetration effect compared to visible light, (2) the induced thermal effect may dramatically enhance cell permeability that is helpful for chemo-drug delivery, and (3) phototherapy is minimally invasive and could be less toxic to normal cells/tissues through the use of a targeting photosensitizer and/or spatially controlled light irradiation [22,23]. In general, phototherapy is carried out by hyperthermia and/or reactive oxygen species (ROS) generated by the photosensitizers after light exposure in the presence of oxygen. The former may cause thermal ablation of cancer cells (i.e., photothermal therapy; PTT), while the latter may seriously interfere with cellular metabolism and consequently trigger programmed cell death (i.e., photodynamic therapy; PDT) [24,25]. No matter which anticancer mechanism is utilized, photosensitizer plays the key role in the phototherapy. Moreover, it is crucial to simultaneously deliver both anticancer drugs and photosensitizers with accurate dosages to the tumor site for an effective photochemotherapy.

Indocyanine green (ICG), a water-soluble tricarbocyanine dye which can be absorbed and fluoresce in the region of 650–850 nm, is one of the USFDA-approved NIR fluorophores and has long been used as a contrast agent in broad clinical diagnostics such as optical coherence tomography–angiography [26], fluorescence-guided oncologic surgery [27], and cerebral perfusion diagnosis [28]. In research, ICG is also able to serve as an exogenous fluorophore in FDOT-mediated breast cancer detection and characterization [29,30,31]. Evidently, leaky tumor vasculature indeed delays ICG washout and hence increases its concentration in tumors compared to normal tissues [32,33]. In addition to imaging applications, ICG has also been extensively used for phototherapy, including breast, skin, and liver tumors [34,35,36], since it may generate hyperthermia and singlet oxygen upon NIR irradiation when performing PTT and PDT, respectively. However, quite a few unfavorable properties of ICG such as rapid plasma clearance, poor hydrolytic stability, poor photo and thermal stabilities, and concentration-dependent aggregation [37,38] severely hinder its usability in the clinic.

Nanotechnology may offer a feasible means for co-administration of multiple agents such as ICG and CPT without the aforementioned drawbacks because it may provide enhanced bioavailability, improved stability, and security to the payloads [39]. In this study, we sought to develop ICG and CPT co-loaded perfluorocarbon (PFC) double-layer nanocomposites (ICPNCs) and explore their potentials in photochemotherapy and FDOT diagnosis of breast cancer. PFC, a fluorine-substituted derivative of hydrocarbons, is a known oxygen transporter since it can dissolve large respiratory gases compared to water [40], and therefore it is highly advantageous for use in PDT applications. We anticipate that the developed ICPNC is a robust theranostic nanoplatform which is able to (1) protect the encapsulated ICG from degradation due to external influences such as light and/or heat, (2) serve as an NIR contrast agent for FDOT detection, (3) provide an on-demand cell-binding specificity through surface modification, and (4) offer an enhanced tumoricidal efficacy with reduced chemotoxicity since the dual photochemotherapy may decrease the effective dosage of the anticancer drug used in the chemotherapy alone. In this paper, we first introduce the fabrication of ICPNCs, followed by investigations of their physicochemical properties, surface modifiability for cell targeting, potential for use in FDOT, and anticancer efficacy in vitro and in vivo.

## 2. Materials and Methods

### 2.1. Fabrication, Surface Modification, and Characterization of the ICPNCs

The ICPNCs were fabricated using a modified emulsification approach. In brief, 500 μL of methanol (50% *v/v*) containing CPT (0.02 wt%) and ICG (0.1 wt%) was first mixed with 900-μL perfluorooctyl bromide (PFOB) containing polyethoxylated fluorosurfactant (2 wt%) at ambient temperature. The mixture was sonicated in an ice bath for 5 min to form the primary water-in-PFC (W/PFC) emulsions. The produced emulsions were then immediately added to an aqueous solution containing carboxylic Pluronic F68 copolymer (CP-F68; 5 wt%) which was synthesized using the method reported previously [41], followed by homogenization in an ice bath for 10 min. The obtained ICPNCs with a water-in-PFC-in-water (W/PFC/W) structure were washed twice with deionized water (DIW) and stored at 4 °C until use. 

**Figure 1:**
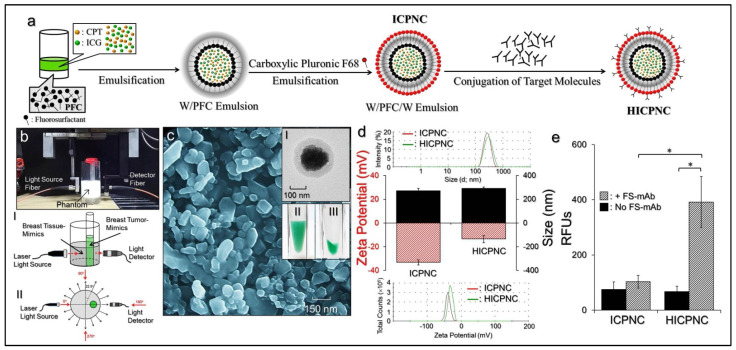
Assessment of physicochemical properties of the ICPNCs and HICPNCs. (**a**) The ICPNCs were synthesized through double emulsification processes using polyethoxylated fluorosurfactant and CT-F68 as the emulsifiers. The HICPNCs were obtained after conjugation with anti-HER2-mAb on the ICPNC surface. (**b**) Photograph of the phantom setup using 1S-1D scanning platform. The schematic drawings I and II display the relative positions of the BTiM, BTuM, laser light source, and light detector from side (I) and top (II) views, respectively. (**c**) SEM image of the ICPNCs at 15,000× magnification. The inset photographs exhibit the TEM image (20,000×) of the ICPNC (I) and the real appearance of the ICPNC sample before (II) and after (III) centrifugation. (**d**) Size and surface charge (ζ—potential) distribution profiles of the ICPNCs and HICPNCs measured by DLS. Values are mean ± s.d. (*n* = 3). (**e**) Verification of the presence and bioactivity of anti-HER2-mAb on the ICPNC surface. The intensity of fluorescence expressed from each group was measured by spectrofluorometry performed with 488/525 nm of excitation/emission wavelength and was quantitatively represented by RFUs. Values are mean ± s.d. (*n* = 3). * *p* < 0.05.

The conjugation of anti-HER2-monocoloal antibody (mAb) on the ICPNC surface was conducted using the carboxyl-amine crosslinking reaction. Briefly, the ICPNCs were first reacted with *N*-(3-dimethylaminopropyl)-*N’*-ethylcarbodiimide hydrochloride (EDC) and sulfo-*N*-hydroxysuccinimide (Sulfo-NHS) with a molar ratio of EDC/sulfo-NHS = 9:1 in PBS at 4 °C for 2 h. After washing twice with PBS, the carboxyl-activated ICPNCs were mixed with 100 μg of anti-HER2-mAb and maintained at 4 °C for 16 h. Afterward, the synthesized anti-HER2-mAb-conjugated ICPNCs (HICPNCs) were washed with DIW and lyophilized. The fabrication procedures of the ICPNCs and the HICPNCs are illustrated in Figure 1a. 

The size distribution and surface charge of the ICPNCs and HICPNCs were measured using dynamic light scattering (DLS). The morphology of the ICPNCs was detected using both scanning electron microscopy (SEM) and transmission electron microscopy (TEM) with negative staining of phosphotungstic acid (PTA; 2%). The encapsulation efficiency (*E*) of the drug (CPT or ICG) was evaluated using the formula:

(1) $$E=\frac{W_{o}{\;-\;W}_{s}}{W_{o}}\times 100\%$$

where *W*_o_ is the amount of ICG or CPT originally used for the ICPNC fabrication and *W*_s_ denotes the amount of unencapsulated drug molecules in the supernatant. Both *W*_o_ and *W*_s_ were determined by spectrophotometry (λ = 370 nm for CPT; 780 nm for ICG) according to Beer–Lambert’s law. The loading rate (*L*; wt%) of the payload (CPT or ICG) in the ICPNCs was evaluated using the formula:

(2) $$L=\frac{W}{W_{\mathrm{NC}}}\times 100\%$$

where *W*_NC_ is weight of the nanocomposites and *W* denotes the weight of the CPT or ICG loaded in the nanocomposites (~*W*_o_ × *E*). 

The presence and bioactivity of the anti-HER2-mAb on the ICPNC surface were identified by using the fluorescent secondary antibody (FS-mAb) as the probe. The fluorescence levels expressed from the FS-mAb-conjugated nanocomposites (ICPNCs or HICPNCs) were detected by spectrofluorometry performed with 488/525 nm of excitation/emission wavelength. In this study, the intensity of fluorescence was quantitatively represented by relative fluorescence units (RFUs) and was analyzed after normalization against the background signal.

### 2.2. Evaluation of Stability and Drug Release Efficiency of the ICPNCs

Both the thermal stability of the encapsulated ICG and the release kinetics of the loaded CPT were analyzed in this study. After incubation in PBS at 4 or 37 °C for 2, 4, 12, 24, and 48 h, the ICPNCs and their supernatant collected by centrifugation were separately subjected to spectrophotometry to measure the quantity of ICG remaining in the nanocomposites (λ = 780 nm) and the amount of CPT released from the nanocomposites (λ = 370 nm), respectively.

### 2.3. Measurement of ICPNC-Induced Hyperthermia Effect

To evaluate the potential of the ICPNCs for use for PTT, ICPNCs containing 0, 2, 4, 20, 40, or 80 μM of ICG in 200-μL DIW were irradiated by an 808-nm laser with an intensity of 6 W/cm^2^, whereby the temperature of each group was recorded every 30 s for 5 min using a digital thermometer. 

### 2.4. Measurement of Yield of ICPNC-Induced Singlet Oxygen 

The productions of singlet oxygen generated from the ICPNCs under NIR laser exposure (808 nm; 6 W/cm^2^) were measured using the commercial singlet oxygen sensor green (SOSG) kit (Life Technologies, Carlsbad, CA, USA) according to the manufacturer’s instructions. The concentrations of encapsulated ICG were set as 0, 2, 4, 20, 40, and 80 μM. The level of SOSG-induced fluorescence in each group was measured by spectrofluorometry every 60 s for 5 min and was quantitatively represented by RFUs. 

### 2.5. Cell Culture

The MDA-MB-453 cells (ATCC^®^ HTB-131^™^, ATCC, Rockville, MD, USA) were cultured in Leibovitz’s L-15 medium supplemented with 10% fetal bovine serum (FBS), 2-mM l-glutamine and 100-U/mL penicillin/streptomycin at 37 °C without CO_2_. BT-474 cells (ATCC^®^ HTB-20^™^) were cultivated using Hybri-Care medium supplemented with 30 ng/mL EGF, 10% FBS, and 100-U/mL penicillin/streptomycin at 37 °C with 5% CO_2_. MDA-MB-231 cells (ATCC^®^ HTB-26^™^) were cultured in DMEM supplemented with 10% FBS and 100-U/mL penicillin/streptomycin at 37 °C with 5% CO_2_.

### 2.6. Assessment of Cell Binding Specificity of the HICPNCs In Vitro

The binding specificity of the HICPNCs was examined by measuring the binding efficiency of the HICPNCs to HER2-expressing breast cancer cells in the presence and absence of competitive mAbs. Briefly, 2.5 × 10^6^ BT-474 cells were aliquoted into five wells of a 24-well culture plate and maintained at 37 °C for 24 h. Afterward, the cells were separately treated with no nanocomposite (blank), ICPNCs, and HICPNCs in the presence of 0, 1, or 2 μg/mL of free anti-HER2-mAbs at 37 °C for 4 h, followed by washing twice with PBS. The intensities of ICG-derived fluorescence expressed from the cells were measured using spectrofluorometry performed with 750/838 nm of excitation/emission wavelength. In this study, the cell-binding efficiencies of the HICPNCs were quantitatively analyzed using the normalized RFUs against the blank control.

### 2.7. Cytotoxicity of the ICPNCs In Vitro

The photochemotherapeutic effect of the ICPNCs on breast cancer was examined using MDA-MB-453 and BT-474 cells as the model cells. For each type of the cell, 1 × 10^5^ cells/well were separately treated with NIR, free CPT, free ICG + NIR, and ICPNCs ± NIR under different ICG/CPT dosages. For the groups with NIR irradiation, the cells together with designated agents were exposed to an 808-nm laser with an intensity of 6 W/cm^2^ for 5 min, followed by maintenance at 37 °C for 24 h. For the groups without NIR treatment, the cells were directly subjected to viability analysis after incubation in the presence or absence of reagents for 24 h. In this study, the concentrations of free ICG and free CPT ([ICG]/[CPT]) were set as 0/0, 2/0.375, 4/0.75, 20/3.75, 40/7.5, and 80/15 μM which were equal to the dosages provided by the ICPNCs. The cell viability of each group was analyzed using MTT and calcein-AM/propidium iodide staining assays.

### 2.8. Experimental Setup of FDOT

The overall FDOT instrumental setup is illustrated in Supplementary Materials Figure S1. Briefly, the optical data measurement for reconstruction of FDOT images consists of two sections including frequency-domain (FD) measurement and continuous-wave (CW) emission measurement. In the FD measurement, the information of attenuated amplitude and delayed phase was acquired by using a photoelectrical measurement module equipped with an intensity-controlled NIR light set as 780/830 nm excitation (*λ*_x_)/emission (*λ*_m_) wavelength. The source light modulated by a function generator at 20 MHz first passed through an excitation filter (transmission rate = 90% at *λ*_x_ = 672–800 nm, FF01-736/128-25, Semrock Inc., Rochester, NY, USA) and was delivered to the detecting objective (e.g., phantom) as shown in Figure S1A. The transmitted light was then detected/collected by an IR-sensitive photomultiplier tube (PMT, H11461-03, Hamamatsu Inc., Hamamatsu City, Japan) mounted at the other side of the detecting objective. A coded signal demodulation element heterodyned the frequencies of measured/reference signals from 20/20.001 MHz to 40.001 MHz/1 kHz, whereby the attenuated light and phase delay information could be characterized from the 1 kHz signature. In the CW emission measurement, a spectrometer (FLAME-S, Ocean Optics, Largo, FL, USA) was employed to count the out-emitted photons for both excitation and emission wavelength. Furthermore, to avoid over-estimation of the fluorescence yield rate due to overloading of the light signals in the spectrometer, an emission filter (transmission rate = 93% at *λ*_m_ = 822–953 nm, FF01-888/131-25, Semrock Inc.) was employed to reduce the lighting power as shown in Supplementary Materials Figure S1A. All the processes, including signal acquisition, PMT manipulation, and parameter setting, were conducted using LabVIEW^®^ (LabVIEW 2020) as the operational interface. 

A liquid phantom simulating the optical properties of normal breast tissue (breast tissue mimic; BTiM) was prepared by using Lipovenoes (20%, Fresenius Kabi, Bad Homburg, Germany), India ink (0.2%), and water with a volume ratio of 22/1/190 in a glass cylinder (V = 50 mL, d = 4.5 cm) and that showed 0.01 mm^−1^ of absorption coefficient (*µ*_a_) and 1 mm^−1^ of scattering coefficient (*µ*_s_) according to the Monte Carlo method [42]. The breast tumor mimic (BTuM) was prepared by using Lipovenoes (20%), India ink (0.2%), and water with a volume ratio of 14/1/53 (*µ*_a_ = 0.02 mm^−1^; *µ*_s_ = 2 mm^−1^) in a single glass capillary (V = 1 mL, d = 10 mm) that was embedded in the BTiM. The phantom setup is illustrated in Figure 1b. In this study, different conditions of free ICG solutions or ICPNCs were separately employed as the NIR contrast agents for FDOT and the experimental design is presented in Table 1. 

The ICG-treated groups showing different ICG distribution ratios in the whole phantom system (*R*_I_) were set to mimic off-target characteristics of the free ICG molecules, while the ICPNCs were only placed in the BTuM due to assumed cell-binding specificity of the nanocomposites.

(3) $$R_{I}=\frac{V_{\mathrm{FTu}}}{V_{0}}\times 100\%$$

where *V*_FTu_ is the volume of the fluorophore applied to the BTuM and *V*_0_ denotes the total volume of the fluorophores used for the phantom preparation. In this study, the optical data measurement was performed by using a one-source-one-detector (1S-1D) scanning platform where the source light was launched at the cylindrical BTiM every 22.5° so that a total of 16 irradiation positions were set up as shown in Figure 1(bII). For each irradiation, the transmitted light was separately detected from the other 15 positions (except the one for source light) that resulted in a total of 240 datapoints for both intensity and phase difference for each round of phantom detection. 

### 2.9. FDOT Modeling

The FDOT technique is a model-based inverse approach that aims to find the intensity of the fluorophore within a scattering material. In this study, the algorithms of NIR fluorescence imaging reconstruction were straightforward expansions of the methods originally developed for the DOT since the characteristics of the photon density waves diffusing in the tissue can be modeled by the same forward model and the diffusion equations. In a high scattering medium with fluorophores, the fluorescence light generated from the fluorophores excited by an external light source (*S*) can be modeled by the following three diffusion equations [43]:

(4) $$\nabla \cdot D_{x}\left(r\right)\nabla \Phi_{x}\left(r,\omega \right)-\left[\mu_{a_{x}}\left(r\right)-\frac{i\omega }{c}\right]\Phi_{x}\left(r,\omega \right)=-S\left(r,\omega \right)$$

(5) $$\nabla \cdot D_{m}\left(r\right)\nabla \Phi_{m}\left(r,\omega \right)-\left[\mu_{a_{m}}\left(r\right)-\frac{i\omega }{c}\right]\Phi_{x}\left(r,\omega \right)=-S\left(r,\omega \right)$$

(6) $$\nabla \cdot D_{m}\left(r\right)\nabla \Phi_{m}\left(r,\omega \right)-\left[\mu_{a_{m}}\left(r\right)-\frac{i\omega }{c}\right]\Phi_{m}\left(r,\omega \right)=-\Phi_{x}\left(r,\omega \right)\eta \mu_{af}\left(r\right)\frac{1+i\omega \tau \left(r\right)}{1+{\left[\omega \tau \left(r\right)\right]}^{2}}$$

where the subscripts *x* and *m* denote excitation and emission, respectively, *τ* is fluorescence lifetime, *ημ_af_* is fluorescence yield, and *c* is the speed of light in the medium. *μ*_a_(**r**) and *D*(**r**) represent the absorption and diffusion coefficient at position **r**, respectively, in which the *D*(**r**) is related to the reduced scattering coefficient *μ*_rs_ defined by *D*(**r**) = 1/[3(*μ*_a_(**r**) + *μ*_rs_(**r**))]. Φ(**r**,*ω*) represents the photon density at position **r** with light modulation frequency *ω*. In this study, both photon densities of excitation light (Φ*_x_*) and emission light (Φ*_m_*) were solved using the finite-element method under known optical properties of the phantom as reported previously [44]. In addition, the Robin boundary conditions (type-III) were used to account for the refractive index mismatch between the phantom and the surrounding medium [45].

### 2.10. Assessment of Effect of ICPNC-Aided FDOT In Vitro

In this study, the effects of FDOT with different conditions of detection of BTuM were quantitatively assessed by the tumor resection quality index (TRQI). The TRQI represents the possibility to remove the most tumor tissues but minimal normal breast tissues of the tomographic setting and is defined by

(7) $$\mathrm{TRQI}=\frac{R_{\mathrm{RST}}}{R_{\mathrm{RSN}}}-\frac{R_{\mathrm{RMT}}}{R_{\mathrm{RMN}}}$$

where *R*_RST_, *R*_RMT_, *R*_RSN_, and *R*_RMN_ denote the percentages of the resected tumor, the remaining tumor, the resected normal breast tissue, and the remaining normal breast tissue, respectively. The method to calculate each ratio is introduced in detail in Supplementary Materials Figure S2. The FDOT algorithm was calibrated based on the known BTuM position in the whole phantom system. In this study, the TRQIs of seven FDOT groups (Table 1) and one DOT group were analyzed based on the reconstructed images obtained.

### 2.11. Animal Model

In this study, BALB/c nude mice (8–12 weeks, female) weighing 25–35 g were employed to build up the in vivo breast cancer model. All animal procedures including the care and operation of laboratory animals were in accordance with the guidelines approved by the Cathay General Hospital (Taiwan ROC, approval number: IACUC110-002). The tumor xenografts were implanted in the BALB/c nude mice by injecting 5 × 10^6^ MDA-MB-231 cells into the flank region of each mouse. Tumor volume was calculated by (*L* × *W*^2^)/2 where *L* and *W* denote the tumor length and width, respectively.

### 2.12. Evaluation of Retention Effect of the ICPNCs in Tumor

To evaluate the tumor retention effect of the ICPNCs in vivo, six tumor-bearing nude mice were intratumorally injected with 150-μL free ICG (*n* = 3) or ICPNCs (*n* = 3) and both agents contained 100 μM of ICG. The ICG-derived fluorescence signals expressed from the injected tumors were detected using an in vivo imaging system (IVIS) (IVIS^®^ Spectrum, PerkinElmer, Waltham, MA, USA) performed with 760/830 nm of excitation/emission wavelength. All mice were anesthetized via isoflurane inhalation (3–5% isoflurane accompanied with 1.5 L/min of oxygen) prior to and during the retention examination. The fluorescence images of each mouse were collected by the IVIS imager beginning at 30 s post injection and the detection lasted for seven days. The fluorescence intensities in the region of the tumor were analyzed using Living Image 3.0 and were quantitatively represented by RFUs.

### 2.13. Evaluation of Anticancer Efficacy of the ICPNCs In Vivo

To evaluate the anticancer effect of the ICPNCs in vivo, the nude mice bearing MDA-MB-231 tumors were intratumorally injected with 40 μL of PBS (control), free CPT, ICG-loaded PFC nanocomposites (IPNCs), or ICPNCs, and treated with and without NIR as indicated in Table 2. In this animal study, the NIR irradiation was performed using an 808-nm laser with an output intensity of 6 W/m^2^ for 60 s. The concentrations of ICG and CPT in the settings of IPNCs and/or free CPT were equal to the dosages provided by the ICPNCs and they were determined based on the results of cytotoxicity of the ICPNCs in vitro. Each treatment was performed every three days for a total of 21 days. The tumor sizes, tumor appearances, and body weights of the tumor-bearing mice were measured at days 0, 3, 6, 9, 12, 15, 18, and 21 before treatment. The experimental animals were sacrificed at the 21st day, then the tumors and different organs were excised for subsequent histological and biocompatibility analyses. Considering that the mice may die during the experiment, in total 120 mice were prepared in this animal study. For the analyses of tumor size and body weight variations, the data of each group were presented based on the results of the 5 mice which were alive throughout the animal study (i.e., lived for 21 days before sacrifice). The survival rate of each group was calculated based on the total live/dead numbers of mice employed for obtaining 5 live mice on the 21st day.

### 2.14. Histological Study

For each experimental mouse, five organs, the heart, liver, spleen, lung, and kidney, and the skin tissue around the tumor were harvested by sharp dissection and weighed immediately after sacrifice. All tissue specimens were fixed by formalin (fixation time = 12 h) and dehydrated in graded ethanol (≥90%, *t* ≥ 15 min six times), followed by xylene clearance (*t* ≥ 20 min three times), wax infiltration (*t* ≥ 30 min three times), and paraffin embedding for a routine histological process. A 5-μm section obtained from each paraffin block was processed with hematoxylin and eosin (H&E) and interlukin-1 beta (IL-1b) immunohistochemical (IHC) stainings, followed by microscopic analysis using Motic DSA software (Motic, Kowloon, Hong Kong).

### 2.15. In Vivo Biocompatibility Evaluation

To assess the in vivo systematic toxicity of each treatment, blood of each mouse was collected at two time points: (1) 7 days before drug treatment (day −7) and (2) the time before sacrifice (day 21), and immediately subjected to blood biochemistry analysis where the expression levels of glutamic oxaloacetic transaminase (GOT), glutamic pyruvic transaminase (GPT), blood urea nitrogen (BUN), and creatinine (CRE), as well as numbers of white blood cells (WBCs), red blood cells (RBCs), and platelets (PLTs), were quantitatively measured using a blood analyzer (FUJI DRI-CHEM 4000i, FUJIFILM, Tokyo, Japan). For the nude mice treated with PBS, CPT, ICPNCs, and ICPNCs + NIR, the amounts of CPT remaining in their five organs and tumors were additionally measured using a spectrophotometer at λ = 370 nm.

### 2.16. Statistical Analysis

All data were acquired from ≥three independent experiments and are presented as the mean ± standard deviation (s.d.). Statistical analyses were conducted using MedCalc software (Version 17.2) in which comparisons for one condition between two groups were performed by Student’s *t*-test with a significance level of *p* < 0.05 throughout the study.

## 3. Results and Discussion

### 3.1. Characterization of the ICPNCs and HICPNCs

Figure 1c exhibits the SEM image of the ICPNCs where it can be seen that the produced ICPNCs remained in an intact particulate configuration without collapse after the manufacturing procedures, including high-speed centrifugation, agitation, and lyophilization, while the double-layer structure of the nanocomposite can be observed through PTA-staining TEM (Figure 1(cI)). The ICPNC sample displays a green-to-yellow emulsified appearance as shown in Figure 1(cII). The colored sediment shown in Figure 1(cIII) indicates that the ICG and CPT were certainly entrapped in the nanocomposites rather than freely dissolved in the medium. According to the DLS analysis, the surface charge and the size of the ICPNCs were −33.3 ± 1.8 mV and 271.8 ± 24 nm, respectively (Figure 1d). Their negative charge is possibly due to a plethora of carboxylic moieties on the emulsion surface. The encapsulation efficiencies of ICG and CPT in the ICPNCs were 98.12 ± 1.3% and 40.3 ± 7.6%, while their loading rates were about 0.34 ± 0.12 wt% and 0.12 ± 0.05 wt%, respectively.

The HICPNCs exhibited a zeta potential of −13.3 ± 3.1 mV and a mean size of 292.2 ± 12 nm as plotted in Figure 1d. The relative neutral surface and enlarged size compared to those in ICPNCs were attributed to the presence of anti-HER2-mAbs on the nanocomposite surface. According to the results of secondary antibody assay, the fluorescence expression level of the HICPNCs with FS-mAbs was 5.8-fold (*p* < 0.05) and 3.8-fold (*p* < 0.05) higher than the values obtained from the HICPNCs without FS-mAbs and the FS-mAbs-treated ICPNCs, respectively (Figure 1e), indicating that the anti-HER2-mAbs were certainly bound on the ICPNC surface and can provide an intact bioconjugatability to the target molecules.

### 3.2. Thermal Stability of ICPNC-Encapsulated ICG and Efficiency of CPT Release

**Figure 2:**
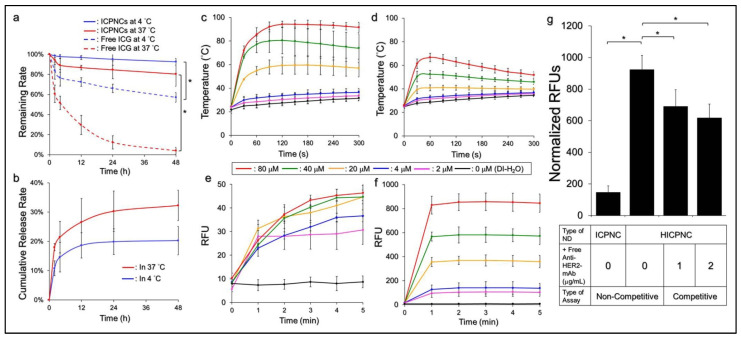
Assessment of stability and functionality of the ICPNCs in vitro. (**a**) Quantitative analysis of ICG remaining in the ICPNCs or PBS under incubation at 4 or 37 °C for 48 h. (**b**) Cumulative release profiles of the ICPNC-encapsulated CPT under incubation at 4 or 37 °C in PBS for 48 h. (**c**,**d**) Temperature elevation profiles of freely dissolved ICG (**c**) and the ICPNCs (**d**) under NIR irradiation for 5 min. (**e**,**f**) Singlet oxygen productions of freely dissolved ICG (**e**) and the ICPNCs (**f**) under NIR irradiation for 5 min. The yield of singlet oxygen was assessed based on the intensity of the SOSG-induced fluorescence measured by spectrofluorometry performed with 488/525 nm of excitation/emission wavelength and quantitatively represented by RFUs. In (**c**–**f**), groups with different ICG concentrations are denoted by different line colors as indicated in the figure. NIR irradiation was performed using an 808-nm laser with an output intensity of 6 W/cm^2^ for 5 min. (**g**) Verification of HER2-binding specificity of the HICPNCs. The histogram shows the fluorescence levels of the BT-474 cells after treatment with ICPNCs or HICPNCs in the presence (competitive; 1 or 2 μg/mL) and absence (non-competitive) of free anti-HER2-mAb for 4 h. The ICPNCs and HICPNCs containing 40 µM ICG and 7.5 µM CPT were employed for the experiment. The intensities of ICG-derived fluorescence were measured by spectrofluorometry performed with 750/838 nm of excitation/emission wavelength and were quantitatively represented by RFUs. Values in (**a**–**g**) are mean ± s.d. (*n* = 3). * *p* < 0.05.

Figure 2a shows the degradation profiles of the ICPNC-entrapped ICG and freely dissolved ICG under incubation at 4 or 37 °C in the dark within 48 h. Based on the OD_780_ analysis, the remaining percentages of ICG in the nanocomposites were significantly raised 1.6-fold (*p* < 0.05) and 20-fold (*p* < 0.05) compared to those in the PBS after 48 h at 4 and 37 °C, respectively, indicating that the thermal stability of ICG was dramatically enhanced after being entrapped in the ICPNCs.

The measurements of the release efficiency of CPT from the ICPNCs at 4 and 37 °C were concurrently performed with detection of ICG degradation. As shown in Figure 2b, both groups exhibited a biphasic release profile that consisted of an initial burst release followed by a sustained slow release for the encapsulated CPT, showing a release rate of 20.3 ± 4.88% and 32.3 ± 5.22% for incubation at 4 and 37 °C, respectively, after 48 h. We speculate that the increased release rate for the group at 37 °C resulted from promoted demulsification of the ICPNCs such as coalescence, Ostwald ripening, and/or phase inversion/separation of the nanocomposites [46] since temperature elevation may increase Brownian motion of particles with d < 2 μm [47] and therefore the collision of the ICPNCs accelerated which led to enhanced collapse of nanocomposites and rapid CPT release, accordingly.

### 3.3. Effects of Hyperthermia and Singlet Oxygen Production of the ICPNCs

Figure 2c,d show the effects of hyperthermia generated from various concentrations of free ICG (Figure 2c) or ICPNCs (Figure 2d) under NIR irradiation for 5 min. Similar to the free ICG, the temperature in each ICPNC group rapidly elevated within the first minute of NIR exposure and was maintained at the same level (settings with ≤20-μM ICG) or slowly declined (groups with ≥40-μM ICG) afterward, yielding an increase of 9.6, 10.9, 11.6, 13.8, 19.7, and 25.7 °C after 5 min NIR irradiation for the ICPNCs set as 0-, 2-, 4-, 20-, 40, and 80 μM ICG, respectively. However, it could be seen that the level of ICPNC-induced temperature elevation was lower than that caused by free ICG under the same concentration setting. This result was explained that, unlike the freely dissolved ICG where all the ICG molecules can simultaneously react to the NIR irradiation, the ICPNC-induced hyperthermia effect was only contributed to by partially released ICG. Moreover, demulsification occurring during NIR exposure is a heat absorption process [48] and that may reduce the thermal energy given to the surroundings. Therefore, the level of ICPNC-induced temperature elevation was relatively mild compared with that performed by the free ICG. Nonetheless, these outcomes clearly showed that the ICPNCs were certainly able to provide a dose-dependent hyperthermia effect upon NIR irradiation.

Similar to the results of hyperthermia examination, our data showed that the ICPNCs can provide a dose-dependent production of singlet oxygen (Figure 2f) upon NIR irradiation as performed by the free ICG (Figure 2e). However, the yield of the former was tremendously higher than that generated from the latter under the same concentration setting. Based on the RFU analysis, the ICPNCs were able to provide 3.3-, 3.7-, 8.1-, 12.8-, and 18.3-fold (*p* < 0.05 for each) larger amounts of singlet oxygen than the free ICG after 5 min NIR irradiation (808 nm, 6 W/cm^2^) when the dose of ICG was set as 2-, 4-, 20-, 40-, and 80 μM, respectively. These results showed that the ICPNCs were indeed able to offer an increased production of singlet oxygen compared to the same concentration of free ICG upon NIR exposure, and such enhanced ROS productivity was reasonably explained due to high oxygen dissolubility of PFC [40] in the ICPNCs.

### 3.4. Binding Specificity of the HICPNCs In Vitro

Figure 2g shows the level of ICG-derived fluorescence expressed from the BT-474 cells after treatment with ICPNCs or HICPNCs in the presence and absence of competitive antibody for 4 h. Based on the RFU analysis, the fluorescence level of the HICPNC-treated cells was 6.3-fold (*p* < 0.05) higher than that obtained from the ICPNC-treated cells in the absence of competitive molecules. We reason that the binding of the HICPNCs to the HER2-expressing BT-474 cells was achieved by receptor-mediated endocytosis, while that of the ICPNCs was simply carried out through adsorption, the normal method for cancer cells to internalize negatively charged nanoparticles as reported previously [49]. Since receptor-mediated endocytosis is more efficient and specific than adsorption [50], it is reasonable that the HICPNCs can rapidly bind with the BT-474 cells and conferred to them significantly enhanced RFUs compared to those with ICPNCs. To further ensure that the increased binding was achieved through conjugation with cellular HER2 receptors, the ICG-derived fluorescence expression of the BT-474 cells after being co-cultured with the HICPNCs and competitive anti-HER2-mAbs was examined. As shown in Figure 2g, the RFUs significantly decreased 25.2% (*p* < 0.05) and 33.1% (*p* < 0.05) when the dose of free anti-HER2-mAbs was set as 1 and 2 μg/mL, respectively, indicating that the binding of the HICPNCs to the HER2-expressing cells was certainly affected by the availability of HER2 receptor–ligand conjugation. These results suggest that the surface of the ICPNC is modifiable and the developed nanocomposites can be equipped with cell-binding molecules to provide on-demand breast cancer targetability.

### 3.5. Cytotoxicity of the ICPNCs In Vitro

**Figure 3:**
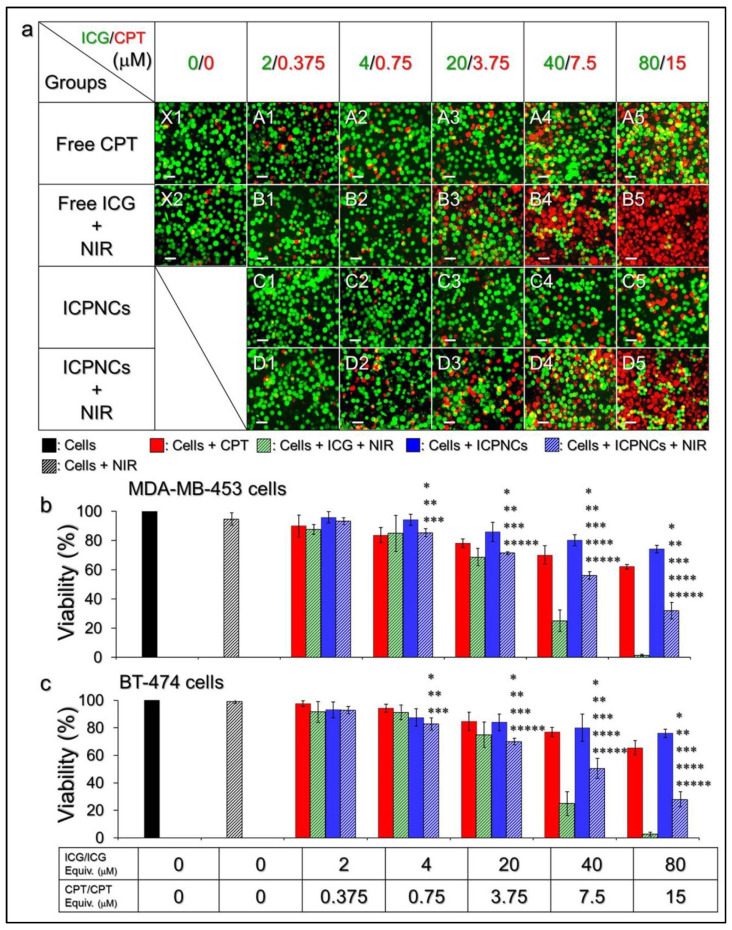
Cytotoxicity of the ICPNCs to breast cancer cells in vitro. (**a**) Photomicrographic images of MDA-MB-453 cells under various treatments. Rows A–D represent the groups treated with CPT, free ICG + NIR, ICPNCs, and ICPNCs + NIR, respectively, in which the concentrations of ICG and/or CPT (ICG/CPT) were set as 2/0.375, 4/0.75, 20/3.75, 40/7.5, and 80/15 μM as indicated in the figure. NIR irradiation was performed using an 808-nm laser with an output intensity of 6 W/cm^2^ for 5 min. ×1 denotes the cells with neither drug nor NIR exposure. ×2 represents the cells treated with NIR exposure for 5 min followed by incubation at 37 °C for 24 h. The green and red cells stained by calcein-AM and propidium iodide represent live and dead cells, respectively. All images were photographed using a fluorescence microscope at 200X magnification. Scale bar = 30 μm. (**b**,**c**) Quantitative analyses of the viabilities of MDA-MB-453 (**b**) and BT-474 (**c**) cells after treatment with different conditions as indicated on the X-axis. Values are mean ± s.d. (*n* = 3). * *p* < 0.05 compared to the group without treatment. ** *p* < 0.05 compared to the group with NIR exposure alone. *** *p* < 0.05 compared to the group with equal concentration of free CPT. **** *p* < 0.05 compared to the group with free ICG + NIR under equal concentration setting. ***** *p* < 0.05 compared to the group with equal dose of ICPNCs without NIR irradiation.

Figure 3a shows the living/dead conditions of MDA-MB-453 cells after being treated with various doses of ICG, CPT, or ICPNCs with or without NIR exposure. According to the MTT analyses (Figure 3b), >95% of the cells survived after being treated with NIR exposure (Figure 3a, ×2), indicating that slight temperature rises by NIR irradiation without ICG (Figure 2c) were nontoxic. On the other hand, a dose-dependent cytotoxicity could be obtained for each drug-treated group (Figure 3b). Our data showed that when the [ICG]/[CPT] was set as ≥20/3.75 μM, the cells with ICPNCs + NIR (Figure 3a; D3–D5) suffered a significantly higher mortality compared to those treated by the ICPNCs without NIR exposure (Figure 3a, C3–C5) and/or CPT alone (Figure 3(aA3–aA5)), while the free ICG + NIR may provide the highest efficacy of cell eradication among all groups (Figure 3(aB3–aB5)). Similar results could be found in BT-474 cells as shown in Figure 3c. These outcomes show that the developed nanocomposites are certainly effective for cancer cell eradiation upon NIR irradiation (808 nm; 6 W/cm^2^), but less toxic without NIR exposure. In addition, we found that the cell mortality resulting from the ICPNCs + NIR was even higher than that caused by using double the amount of the encapsulated CPT alone (Figure 3b,c), showing that the phototherapy indeed played a crucial role in ICPNC-mediated anticancer treatment. In addition, although ≥75% of the cancer cells can be killed by using free ICG + NIR with ≥20 μM ICG (Figure 3b,c), it may not be an appropriate approach for use in the clinic due to a number of disadvantages of ICG such as rapid plasma clearance, insufficient stability, and concentration-dependent aggregation [37,38] which are unfavorable for use in vivo. With merits of improved ICG stability, surface modifiability for cell targeting, effective cancer cell eradication, and reduced chemotoxicity, the ICPNC is considered as a more desirable photosensitizer compared to free ICG.

### 3.6. Phantom Study of ICPNC-Aided FDOT Detection

**Figure 4:**
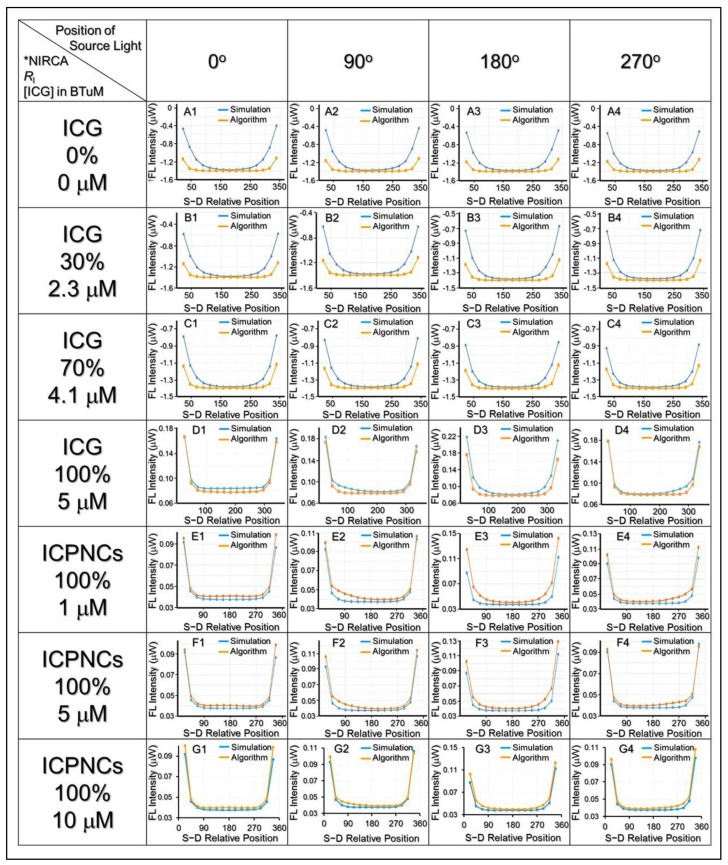
Analyses of FDOT fluorescence signals using free ICG or ICPNCs as the contrast agent in phantom study. Rows A–D represent the groups using free ICG with *R*_I_ = 0%, 30%, 70%, and 100%, while E–G denote the groups using ICPNCs containing 1, 5, and 10 μM of ICG with *R*_I_ = 100% as indicated in the figure. Columns 1–4 denote the positions of the source light as illustrated in Figure 1(bII). The fluorescence intensities expressed from various positions were analyzed using both simulated and algorithmic approaches. The FDOT was performed using an NIR laser with an intensity of 30 mW and the integration time was set as 800 ms. * NIRCA denotes NIR contrast agent. ^†^ FL represents fluorescence.

Figure 4 shows the results of fluorescence intensity expressed from the phantoms with various fluorophore conditions where the detection was performed using a 1S-1D setup as illustrated in Figure 1b. The results show that with an increase in the ICG ratio in the BTuM from *R*_I_ = 0 to 100% (Figure 4A–D), the algorithmic fluorescence intensities were closer to the simulated values that represented an enhanced detection accuracy. Similar results could be found in the groups with *R*_I_ = 100% but various ICG concentrations as shown in Figure 4E–G. Furthermore, it can be seen that the groups with free ICG and ICPNCs containing an equal ICG concentration and *R*_I_ could exhibit similar distribution profiles of fluorescence intensity at each source light position, even though the intensities gained from the former were slightly higher than the latter as shown in Figure 4D,F. These results showed that the ICPNCs can truly serve as an NIR contrast agent for FDOT detection, and the efficacy of ICPNC-aided FDOT was dependent on both the agglomeration degree (i.e., *R*_I_) and the concentration of the contrast agent in the target (e.g., BTuM).

**Figure 5:**
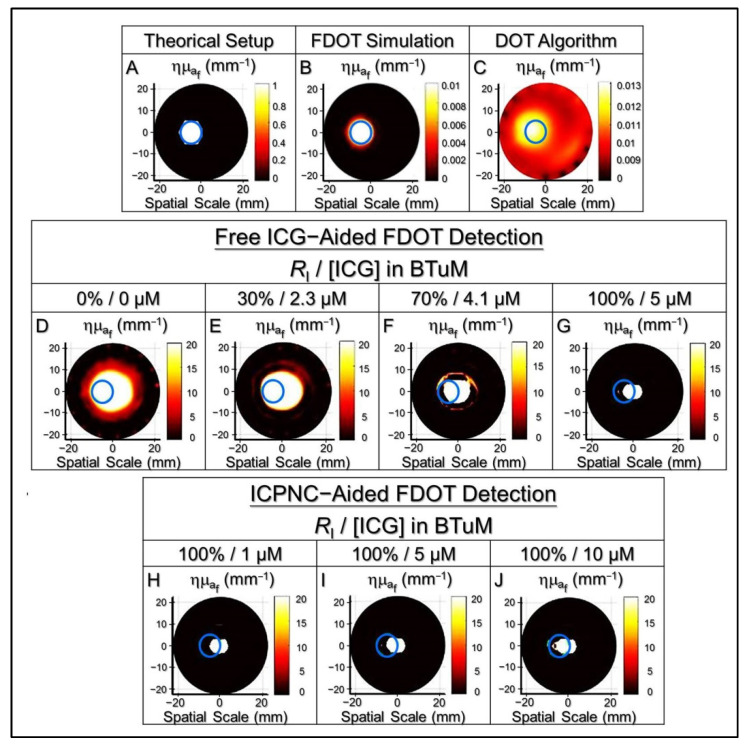
Image reconstructions of BTiM and BTuM using free ICG or ICPNCs as the NIR contrast agent. (**A**–**J**) represent the reconstructed images gained by FDOT or DOT with various conditions as indicated in the figure. The blue circle denotes the true position of the BTuM in each image determined based on the known experimental settings.

All the fluorescent signals were further subjected to image reconstruction through analysis of fluorescence yield distribution in the phantom. As shown in Figure 5, we found that the detected area could be more concentrated and closer to the real BTuM position along with increases of *R*_I_ or concentration of the contrast agent used (Figure 5D–J). Based on the results of image reconstruction, one may notice that the detected zones in groups with *R*_I_ = 0% (Figure 5D) or 30% (Figure 5E) can cover the entire BTuM, while the ones using free ICG with *R*_I_ ≥ 70% (Figure 5F,G) or ICPNCs with *R*_I_ = 100% (Figure 5H–J) cannot. However, the efficacy of the tumor detection should not be merely judged by the fraction of the tumor (BTuM) detected, as from the viewpoint of precision of cancer diagnosis, the percentage of the normal tissue (BTiM) affected by the detection should also be taken into consideration.

In this study, the accuracy and applicability of each setting was determined based on the TRQI that covers the significances of both tumor resection and normal tissue preservation. As presented in Table 3, the TRQI remarkably rose by 12-fold when the *R*_I_ was increased from 0 to 100% in the free ICG settings, while that was enhanced by 6-fold when the ICG concentration in the BTuM was increased from 1 to 10 μM under *R*_I_ = 100% in the ICPNC settings. Among the seven FDOT algorithmic groups (Figure 5D–J), the one with ICPNCs set by 10-μM ICG and 100% of *R*_I_ (Figure 5J) exhibited the highest TRQI (145.01, Table 3) and that was ~145-fold higher than the value gained from the DOT detection (TRQI = 1, Table 3). The group with DOT showed the lowest TRQI compared to all the FDOT algorithmic groups (Table 3). We surmise that it was because DOT is more likely to cause inaccuracy compared to FDOT because DOT is highly susceptible to systematic errors arising from changes in light source–detector coupling and asymmetry in size between tumor and normal tissues [51]. Furthermore, DOT imaging is established based on the different optical coefficients between tumor and normal tissues. Such endogenous contrast can be highly interfered with by adipose tissue and/or dense calcification points near the tumor and thereby led to a deviation of detection [52,53]. Different from DOT, FDOT is carried out through detection of fluorescent signals emitted from the exogenous contrast agents such as ICPNCs in the tumor, therefore, FDOT is able to avoid the aforementioned interferences and serve as a feasible tool for breast tumor detection.

### 3.7. Retention Effect of the ICPNCs in Xenograft Tumor

**Figure 6:**
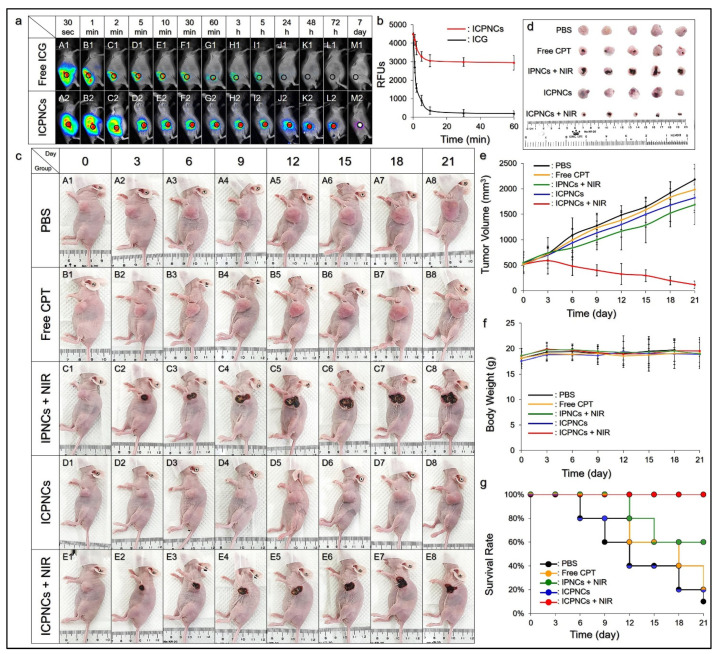
Tumoricidal effect of the ICPNCs in vivo. (**a**) Time-lapse NIR fluorescent images of tumor-bearing mice after intratumoral injection of free ICG (100 μM; A1–M1) or ICPNCs ([ICG] = 100 μM; A2–M2). The black circle denotes the real position of the tumor in vivo. (**b**) Variations of ICG-derived fluorescence expressed from the xenograft MDA-MB-231 tumors injected with free ICG or ICPNCs within 60 min. The intensity of the fluorescence was quantitatively represented by RFUs. Values are mean ± s.d. (*n* = 3). (**c**) Appearances of the MDA-MB-231 tumor-bearing nude mice with treatment of PBS, free CPT, IPNCs + NIR, ICPNCs, or ICPNCs + NIR as indicated in the figure. The condition of the tumor in each mouse was photographed every 72 h for 21 days before treatment or sacrifice. NIR irradiation was performed using an 808 nm laser with an output intensity of 6 W/m^2^ for 60 s. The concentrations of ICG and CPT in the settings of IPNCs and/or free CPT were equal to the dosages provided by the ICPNCs set as [ICG]/[CPT] = 40/7.5-μM. (**d**) Photographs of the tumors harvested after the mice were sacrificed on the 21st day. (**e**–**g**) Variations in tumor size (**e**), body weight (**f**), and survival rate (**g**) of all the experimental mice within 21-day treatment. Values in (**e**,**f**) are mean ± s.d. (*n* = 5). The survival rate (**g**) of each group was calculated based on the total live/dead numbers of mice employed for obtaining five live mice on the 21st day.

Tumors have been known to be able to form a relatively loose vascular network to allow an enrichment of nanomedicine in the neoplastic tissues through the enhanced permeability and retention (EPR) effect [54]. However, a successful nanomedicinal cancer therapy relies on not only the efficiency of drug delivery, but also the effectiveness of drug retention in the tumor. In this study, with confirmed surface modifiability of the ICPNCs for an enhanced/targeting drug delivery effect, we sought to further investigate whether the ICPNCs could stay in the tumor for a longer time. According to the ICG-derived fluorescence expressed from the xenograft tumors (Figure 6a), our data showed that the ICPNCs were indeed able to provide an enhanced drug retention effect compared to free ICG, where the fluorescent signal was even detectable after 7 days (Figure 6(aM2)), while that from the group with free ICG was barely seen after 24 h (Figure 6(aJ1)).

Based on the RFU analysis (Figure 6b), the fluorescence intensity of the group with free ICG dramatically decreased 92% after 10 min, while >65% of that in the group with ICPNCs remained after 60 min. These results clearly showed that the ICPNCs could be accumulated in xenograft MDA-MB-231 tumor for >7 days, and we speculate that such prolonged retention was due to both the tumoral EPR effect and enhanced stability of the encapsulated ICG. On the other hand, a rapid decline in fluorescence for the free ICG was due to its swift molecular aggregation and clearance from the body as reported previously [37,38].

### 3.8. Tumoricidal Effect of the ICPNCs In Vivo

The anticancer effect of ICPNC-mediated photochemotherapy was investigated using xenograft MDA-MB-231 breast tumor-bearing nude mice. Considering that an insufficient antitumor effect or serious tissue damage may occur due to the use of low or high ICG/CPT doses, we selected [ICG]/[CPT] = 40/7.5-μM as the dosage for use in the animal study. Figure 6c shows the conditions of the mice treated by PBS, free CPT, IPNCs + NIR, ICPNCs, and ICPNCs + NIR every three days for a total of 21 days, whereas Figure 6d exhibits the images of the tumors harvested on the 21st day. Based on the analyses of tumor size (Figure 6e), the tumors with free CPT (Figure 6(cB1–cB8)) and ICPNCs without NIR (Figure 6(cD1–cD8)) were dramatically enlarged by 4- and 3.3-fold within 21 days compared to the group with PBS (Figure 6(cA1–cA8)), indicating that those treatments were surely unable to suppress the growth of tumor. IPNCs + NIR may seriously damage the cancer cells which were exposed to NIR (Figure 6(cC1–cC8)), but those without NIR treatment rapidly grew and formed an enlarged tumor with a 3.1-fold increased size after 21 days (Figure 6e). Only ICPNCs + NIR provided an anticancer effect where the tumor growth was dramatically inhibited from day 3 (Figure 6c, E1–E8) and the tumor size was greatly reduced by 80% after 21 days (Figure 6e). In addition, no significant weight loss occurred in all the tumor-bearing mice throughout the time course (Figure 6f), suggesting that the side effect generated by the ICPNCs + NIR with [ICG]/[CPT] = 40/7.5 μM was negligible. Moreover, no tumor recurrence was seen in the group with ICPNCs + NIR throughout the time course (Figure 6e) that conferred a 100% survival rate to the mice after a 21-day treatment (Figure 6g). All the mice were sacrificed on the 21st day, whereby the conditions of tumors showing different sizes after various treatments could be further confirmed (Figure 6d). Taken together, these results clearly demonstrated that the ICPNCs containing [ICG]/[CPT] ≥ 40/7.5 μM in association with 1 min NIR irradiation (808 nm; 6 W/cm^2^) were indeed an effective tumoricidal approach.

In this study, the group with free CPT was employed to mimic the chemotherapy of breast cancer and the concentration was set as 7.5 μM to adapt its dosage provided by the ICPNCs. The ICPNCs alone can provide a higher tumor inhibition effect compared to free CPT according to the result of tumor size measurement (Figure 6e), and that could be attributed to longer retention time of the nanocomposites in the tumor as illustrated in Figure 6a. Nonetheless, this concentration is lower than the half maximal inhibitory concentration (IC_50_) of CPT to MDA-MB-231 cells (~19.2 μM) reported previously [55], and that is why this dosage was not sufficient to suppress tumor growth in vivo. IPNCs with 40-μM ICG may provide both PTT and PDT upon NIR irradiation to eradicate cancer cells as shown in Figure 3. However, without sustained anticancer activity post phototherapy, cells not exposed to NIR remained intact and proliferated with time which yielded a tumor with a significantly increased size after 21 days (Figure 6e). In comparison to the group with ICPNCs + NIR where the tumor size was greatly reduced by 80%, it can be concluded that both photo- and chemotherapeutic functions of the ICPNCs were essential for their use in the anticancer application. Furthermore, such an approach utilizing only 7.5 μM of CPT implied that it not only can provide effective anticancer therapy, but is also able to generate less cancer drug resistance and/or chemotoxicity due to the use of limited CPT.

### 3.9. Inflammatory Response of Skin after ICPNC-Mediated Photochemotherapy

**Figure 7:**
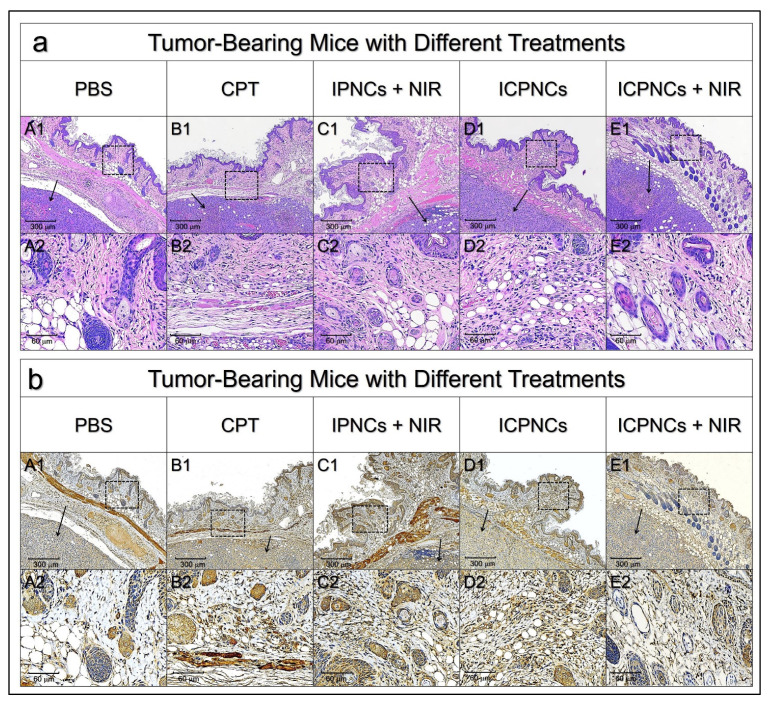
Histological analysis of skin tissue in the vicinity of the tumor. (**a**,**b**) Photomicrographic images of H&E-stained (**a**) or IL-1b IHC-stained (**b**) skin tissues at 4× (A1–E1) and 20× (A2–E2) magnifications. Columns A–E denote different treatments as indicated in the figure. Black arrows in A1–E1 indicate the tumor in the image. The photographs A2–E2 are the magnified images of the area marked by the dashed block in A1–E1.

Figure 7 shows the H&E (Figure 7a) and IL-1b IHC (Figure 7b) staining results of skin tissues in the vicinity of the xenograft tumor. All the groups with drug treatment exhibited increased aggregation of mononuclear cells (Figure 7(aB–aE)) and elevated IL-1b expression (Figure 7(bB–bE)) in the epidermal layer compared to the setting with PBS (Figure 7a,b, column A), showing that an inflammatory response was inevitably induced by photo- and/or chemotherapy. However, interestingly, we found that the inflammation level for the one with ICPNCs + NIR (Figure 7a,b, column E) was relatively mild compared to the other three drug-treated groups. We speculate that this decreased inflammatory response resulted from effective tumor destruction by which the cancer-induced inflammation was reduced [56].

### 3.10. In Vivo Systematic Toxicity Analyses

**Figure 8:**
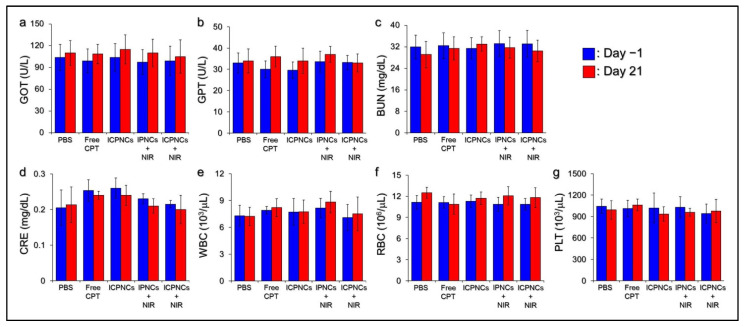
Blood biochemical analyses of the experimental mice. The expression levels of liver (**a**,**b**) and kidney (**c**,**d**) functional markers as well as the numbers of white blood cells (**e**), red blood cells (**f**), and platelets (**g**) of all the experimental mice were measured 24 h before treatment (day −1) and at the moment before sacrifice (day 21). Values are the mean ± s.d. (*n* = 5).

Potential in vivo toxicity is often a critical concern that needs to be addressed for the development of nanomedicine. To assess the medical applicability of ICPNC-mediated photochemo anticancer therapy, the biocompatibility of each method was first evaluated by analyzing the conditions of liver, kidney, and blood cells of the experimental mice during the study. As shown in Figure 8, the expression levels of seven serum markers in the drug-treated groups were all similar to the values gained from the one with PBS throughout the time course, indicating that the treatment of ICPNCs + NIR may not cause liver (Figure 8a,b) or kidney (Figure 8c,d) dysfunction, nor jeopardize blood cells (Figure 8e–g) within the 21-day treatment.

**Figure 9:**
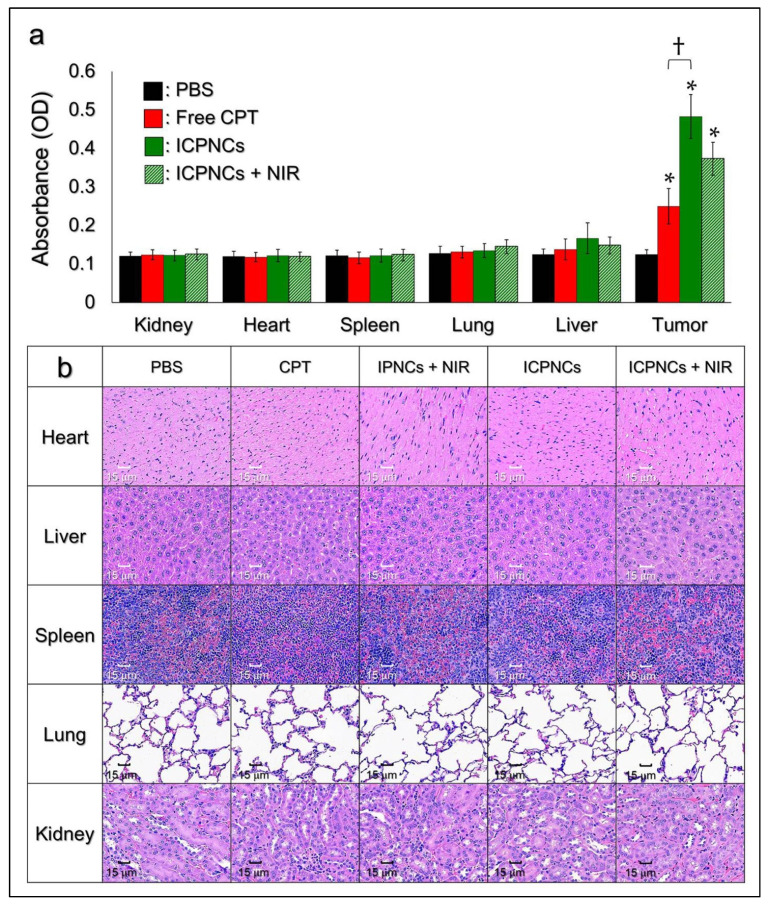
In vivo toxicological study of the ICPNCs. (**a**) Quantitative analyses of the amounts of CPT remaining in the heart, liver, spleen, lung, kidney, and tumor of the MDA-MB-231 tumor-bearing nude mice after treatment with PBS, CPT, ICPNCs, or ICPNCs + NIR for 21 days. Values are the mean ± s.d. (*n* = 5). * *p* < 0.05 compared to the value obtained from the group with PBS for the same organ. ^†^ *p* < 0.05. (**b**) Photomicrographic images of H&E-stained heart, liver, spleen, lung, and kidney obtained from the MDA-MB-231 tumor-bearing nude mice with various treatments for 21 days as indicated in the figure.

The in vivo distributions of CPT from uses of free CPT, ICPNCs, and ICPNCs + NIR were further analyzed after the mice were sacrificed on the 21st day. As shown in Figure 9a, except the tumor where the drugs were directly injected, all five organs exhibited a negligible CPT accumulation regardless of the treatment. In addition, the use of ICPNCs may cause a larger accumulative amount of CPT in the tumor compared with free CPT, and that could be explained by (1) the diffusion rate of nanoagent being lower than free molecules and (2) the encapsulated CPT being protected from enzymatic attack and/or multiple drug clean-up mechanisms such as the reticuloendothelial system and/or transcapillary filtration when the ICPNCs were used in vivo. Nonetheless, neither noticeable injury nor inflammatory lesions were observed in any of the five organs from all of the drug-treated groups according to the toxicological analyses shown in Figure 9b. These results clearly show that ICPNCs + NIR under the designated settings has negligible in vivo toxicity which is highly advantageous for use in the clinic.

## 4. Conclusions

In this study, we have successfully built up a double-layer nanoagent consisting of PFOB, ICG, and CPT (i.e., ICPNC) for detection and treatment of breast cancer. The ICPNCs can protect the entrapped ICG from thermal degradation and offer chemotherapy and photothermal therapy, as well as enhanced photodynamic therapy compared to an equal dose of free ICG upon NIR irradiation. The designed carboxylic surface of the ICPNCs may allow them to be conjugatable with required targeting antibody to achieve on-demand cell binding specificity. In addition, this nanoagent can also be utilized as a NIR contrast agent for FDOT detection and potentially be able to provide an improved cancer diagnosis according to the TRQI analysis. Through the animal study, we further demonstrated that the ICPNCs ([ICG]/[CPT] = 40/7.5-μM) in association with 1-min NIR irradiation (808 nm; 6 W/cm^2^) can provide an exceptional anticancer effectiveness with neither organ damage nor systemic toxicity to the tumor-bearing mice. We reason that such tumoricidal efficacy was achieved by phototherapy followed by chemotherapy; a two-stage anticancer mechanism in vivo. Given the aforementioned efficacies together with another merit of reduced chemotoxicity, we anticipate that the developed ICPNC is highly applicable as a nanotheranostic for both diagnosis and combined treatment of breast cancer in the clinic.

## Figures and Tables

**Table 1:** Experimental setting for the FDOT detection in vitro.

NIRF luorophore	Volume Distribution of ICG in Phantom BTuM:BTiM	Concentration of ICG in BTuM (μM)	*R*_I_
Free ICG *	0:1	0	0
	3:7	2.3	30%
	7:3	4.1	70%
	1:0	5	100%
ICPNC s ^†^	1:0	1	100%
	1:0	5	100%
	1:0	10	100%

* 1-mL ICG solution with 10 μM was used to prepare the fluorescent phantom (BTuM + BTiM). ^†^ 1 mL of ICPNCs containing 0.5, 10, or 20 μM of ICG was used to prepare the BTuM.

**Table 2:** Experimental setting for the in vivo anticancer study.

Reagent Type	* NIR	[ICG] (μM)	[CPT] (μM)
PBS	-	---	---
Free CPT	-	---	^†^ *C*_2_
IPNCs	+	^†^ *C*_1_	---
ICPNCs	-	^†^ *C*_1_	^†^ *C*_2_
	+	^†^ *C*_1_	^†^ *C*_2_

* NIR was performed by an 808-nm laser with an output intensity of 6 W/cm^2^ for 60 s. ^†^ Both *C*_1_ and *C*_2_ were determined based on the results of cytotoxicity of the synthesized nanocomposites in vitro.

**Table 3:** Effect of FDOT detection using free ICG or ICPNCs as the NIR contrast agent in the phantom.

Groups * Factors Area (mm^2^) Ratio	FDOT							DOT
	Free ICG *R*_I_				ICPNCs (*R*_I_ = 100%) [ICG] in BTuM			
	0%	30%	70%	100%	1 μM	5 μM	10 μM	
*A*_T_	78.5	78.5	78.5	78.5	78.5	78.5	78.5	78.5
*A*_D_	580.72	314.29	122.15	46.50	42.96	43.74	51.33	1589.63
*A*_B_	1589.63	1589.63	1589.63	1589.63	1589.63	1589.63	1589.63	1589.63
*A*_NB_	1511.13	1511.13	1511.13	1511.13	1511.13	1511.13	1511.13	1511.13
*A*_RST_	78.5	78.5	60.37	30.27	24.73	31.24	45.33	78.5
*A*_RMT_	0	0	18.13	48.23	53.77	47.26	33.17	0
*A*_RSN_	502.22	235.79	61.78	16.23	18.23	12.5	6	1511.13
*A*_RMN_	1008.90	1275.34	1449.35	1494.89	1492.89	1498.63	1505.13	0
*R*_RST_	100%	100%	76.90%	38.56%	31.5%	39.8%	57.75%	100%
*R*_RMT_	0%	0%	23.1%	61.44%	68.5%	60.2%	42.25%	0%
*R*_RSN_	33.24%	15.6%	4.09%	1.07%	1.21%	0.83%	0.4%	100%
*R*_RMN_	66.76%	84.4%	95.91%	98.93%	98.79%	99.17%	99.6%	0%
TRQI	3.01	6.41	18.57	35.27	25.41	47.5	145.01	1

* All symbols are defined in Supplementary Materials Figure S2.

## Supplementary Materials

The following are available online at https://www.mdpi.com/article/10.3390/pharmaceutics13091499/s1, Figure S1: FDOT equipment setup, Figure S2: Schematic diagram of the related positions between the whole breast tissue (BTiM), breast tumor (BTuM), and the detected region performed by FDOT or DOT.
