# Supplementary material for: Indocyanine Green-Camptothecin Co-Loaded Perfluorocarbon Double-Layer Nanocomposite: A Versatile Nanotheranostics for Photochemotherapy and FDOT Diagnosis of Breast Cancer
Source: Pharmaceutics. 2021 Sep 17;13(9):1499. doi: 10.3390/pharmaceutics13091499 (PMC8466706; doi:10.3390/pharmaceutics13091499)
Supplement: Supplementary file 1 [file pharmaceutics-13-01499-s001.zip › pharmaceutics-1348632-supplementary.pdf]

pharmaceutics

MDPI

Supplementary Materials

Indocyanine Green-Camptothecin Co-loaded Perfluorocarbon Double-Layer Nanocomposite: A Versatile Nanotheranostics for Photochemotherapy and FDOT Diagnosis of Breast Cancer

Yu-Hsiang Lee [1,2,*], Po-Wei Kuo[1,†], Chun-Ju Chen [1,†], Chu-Jih Sue [1], Ya-Fen Hsu [3] and Min-Chun Pan [4,*]

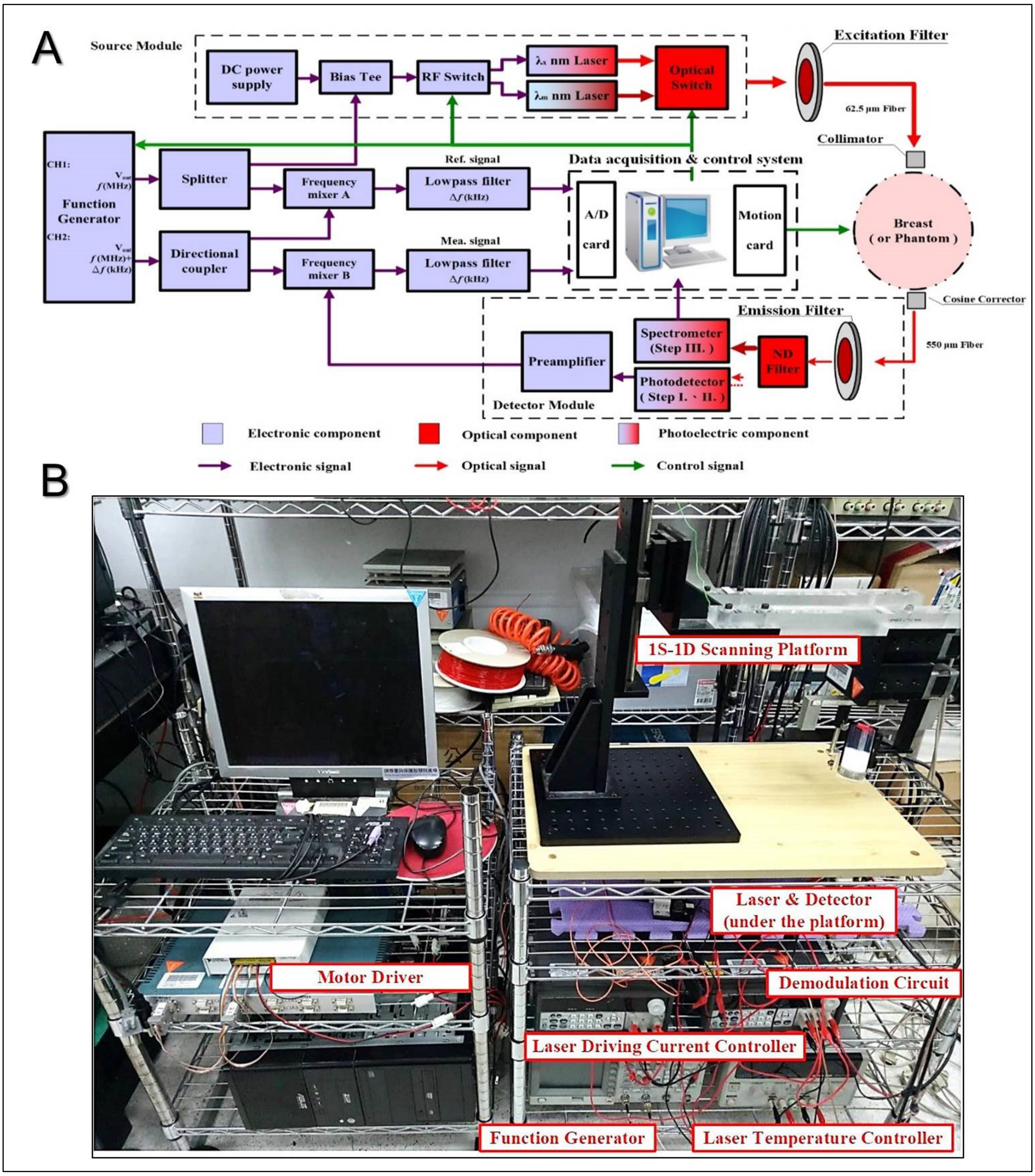

Figure S1. FDOT equipment setup. (**A**) Flow diagram of optical data measurement for FDOT. (**B**) Photograph of FDOT instrumentation.

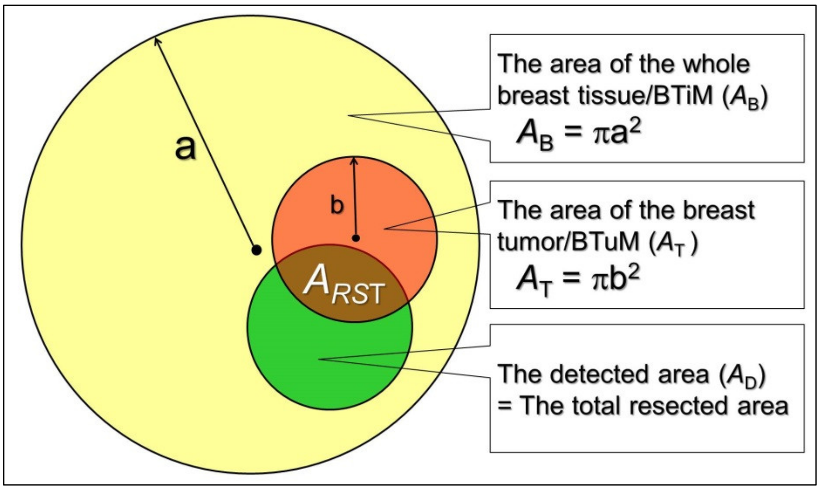

Figure S2. Schematic diagram of the related positions between the whole breast tissue (BTiM; yellow), breast tumor (BTuM; red), and the detected region (green) performed by FDOT or DOT, where the areas are indicated by A_B, A_T, and A_D, respectively. A_{RST} represents the partial tumor area overlapped with the detected region.

S2. Calculations of R_{RST}, R_{RMT}, R_{RSN} and R_{RMN}

The R_{RST}, R_{RMT} , R_{RSN} and R_{RMN}, representing the percentages of the resected tumor, the remained tumor, the resected normal breast tissue, and the remained normal breast tissue, respectively, were calculated based on the hypothesized FDOT imaging outcome shown in Fig. S2. All the areas, except the area of the whole breast tissue (i.e., BTiM, A_B) and the area of the breast tumor (i.e., BTuM; A_T), were measured by ImageJ.

R_{RST}: Percentage of the resected tumor = $\frac{A_{RST}}{A_T}$

R_{RMT}: Percentage of the remained tumor after resection = $\frac{A_{RMT}}{A_T}$

R_{RSN}: Percentage of the resected normal tissue = $\frac{A_{RSN}}{A_{NB}}$

R_{RMN}: Percentage of the remained normal tissue after resection = $\frac{A_{RMN}}{A_{NB}}$

In which all symbols shown above are defined as followings:

A_B: The area of the whole breast tissue (i.e., BTiM) = πa^2 (Fig. S2)

A_D: The area detected by FDOT and that is the total resected area

A_T: The area of the breast tumor (i.e., BTuM) = πb^2 (Fig. S2)

A_{NB}: The area of the normal breast tissue = A_B - A_T

A_{RST}: The area of the breast tumor overlapped with the detected area

A_{RMT}: The area of the remaining breast tumor after resection = A_T - A_{RST}

A_{RSN}: The area of the resected normal breast tissue = A_D - A_{RST}

A_{RMN}: The area of the remaining normal breast tissue after resection = A_{NB} - A_{RSN}
